# Endoscopic healing is associated with a reduced risk of biologic treatment failure in patients with ulcerative colitis

**DOI:** 10.1038/s41598-024-51208-2

**Published:** 2024-01-03

**Authors:** Akira Komatsu, Takahiko Toyonaga, Natsuki Sumiyoshi, Miho Tanaka, Naoki Shibuya, Masayuki Saruta

**Affiliations:** Department of Gastroenterology and Hepatology, The Jikei University School of Medicine, Tokyo, Japan

**Keywords:** Gastrointestinal diseases, Outcomes research, Risk factors

## Abstract

Increasing number of patients with ulcerative colitis (UC) have received biologic treatment during the last decade. The association between endoscopic healing (EH) and biologic treatment failure remains understudied. Medical information of UC patients who started biologic treatment was retrospectively collected. EH was defined as Mayo endoscopic subscore of 0 or 1. Loss of response (LOR)-free drug continuation rate was compared between patients who achieved EH and those who did not using Kaplan–Meier estimator. Fifty-two patients received 53 biologic treatments and underwent follow-up colonoscopies within 2 years. Thirty-three patients achieved EH, all of which remained on the same treatment without LOR during the observational period. Twenty patients did not achieve EH, 8 of which ultimately discontinued the treatment due to LOR to biologic agents. Kaplan–Meier estimator found a significantly lower drug continuation rate in patients without EH (*p* < 0.001; log-rank test). A Cox regression analysis identified EH as an independent factor associated with a reduced risk of LOR-related biologic treatment failure irrespective of the types of biologic agents (Hazard Ratio = 0.0324, *p* < 0.001). EH within 2 years is associated with a reduced risk of LOR-related biologic treatment failure in patients with UC.

## Introduction

Ulcerative colitis (UC) is a chronic inflammatory disorder of the large intestine, which causes relapsing and remitting abdominal symptoms, such as diarrhea, bloody stool, and abdominal pain, in affected patients^1^. Since infliximab (IFX) was approved for the treatment of UC as the first biologic agent by Food and Drug Administration in 2005, an increasing number of biologic agents targeting various inflammatory molecules has been developed. These biologic agents include IFX^2^, adalimumab (ADA)^3^, golimumab (GLM)^4^, vedolizumab (VDZ)^5^, and ustekinumab (UST)^6^ with varying levels of efficacy on the induction and maintenance of remission in the treatment for moderate to severe UC. However, patients often lose response to these drugs over time and require dose intensification or switching to the other types of drugs. Loss of response (LOR) to biologic agents is partially caused by the immunogenicity of drugs which generates anti-drug antibody formation but also occurs in drugs which harbor lower immunogenicity, such as VDZ and UST with unknown mechanisms. A systemic review with meta-analysis found that annual LOR was 10% for IFX and 13% for ADA in patients with UC^7^. The pooled incidence rate of LOR to VDZ was 39.8 per 100 person-years of follow up among patients with UC^8^. Since dose escalation of biologic agents is not approved for UC in many counties including Japan, LOR is one of the most common reasons for discontinuing maintenance treatment with biologic agents. Therefore, there is an unmet need to establish a marker which can help predict LOR and subsequent treatment failure in patients with UC under treatment with biologic agents.

The Selecting Therapeutic Targets in IBD (STRIDE)-II initiative of the International Organization for the Study of IBD (IOIBD) identified endoscopic healing (EH) as a long-term target of UC treatment^9^. EH, also referred to as endoscopic improvement in clinical trials, is defined by Mayo endoscopic sub-score of 0 or 1 and associated with reduced risks of clinical relapse, hospitalization, surgery, and development of colorectal cancer. The achievement of EH is often evaluated as a major clinical outcome in the clinical trials of biologic treatment for UC with varying ranges of achievement rate between 25.0 and 51.6 at 1 year after drug initiation^2–6^. Given the association of EH with preferable clinical outcomes, the absence of EH might be a predictor for LOR-related biologic treatment failure in patients with UC. Nevertheless, their association remains understudied.

In this study, we retrospectively reviewed the disease courses of patients with UC who started biologic treatment and examined the association of EH with LOR-related biologic treatment failure during the maintenance phase of treatment.

## Method

### Patients

A retrospective observational study was conducted on 124 patients with established diagnosis of UC who started anti-tumor necrosis factor (TNF)-alpha agents (IFX, ADA, GLM), VDZ, or UST between January 2018 and December 2022 in the Jikei University Hospital. Thirty-nine patients who stopped biologic treatment within 12 weeks after drug initiation and 33 patients who did not undergo a follow-up colonoscopy after 12 weeks of treatment were excluded from subsequent analyses. Medical records of the remaining 52 patients were reviewed until the end of December 2022.

### Clinical activity score

Clinical activity was evaluated by two-item patient-reported outcome (PRO2) extracted from Mayo score^10^. PRO2-based clinical remission was defined as a stool frequency score of 0 and a rectal bleeding score of 0. Symptomatic remission was defined as a stool frequency score of 0 or 1 and a rectal bleeding score of 0^11^. LOR was defined as disease worsening after primary response to biologic agents, which was evaluated by doctors’ description in medical records. Biologic treatment failure was defined as discontinuation of biologic agents or treatment intensification with other drugs including topical 5-aminosalicylic acid (5-ASA) or corticosteroids for UC due to LOR to biologic agents.

### Endoscopic severity score

Endoscopic severity was evaluated by Mayo endoscopic sub-score (MES). EH was defined as MES 0 or 1 and complete EH was defined as MES 0^9^.

### Statistical analysis

All numeric data are expressed as means ± standard deviation or median ± interquartile range (IQR). Mann–Whitney test was used for the univariate analyses of continuous variables. Fisher exact test or chi-squared test were used for categorical comparisons. *P* values less than 0.05 were considered significant. The Kaplan–Meier method was used to generate survival curves and differences between 2 groups were evaluated by a log-rank test. GraphPad Prism (version 9.5.1 for macOS; GraphPad Software, San Diego, California, USA) was used for these data analyses. A Cox regression analysis were performed using R v4.0.2^12^ and the R package ‘survival’^13^. Sensitivity analyses were performed using 2 explanatory variables which include the presence of EH and one of the following 4 factors: disease extent, concomitant thiopurines, type of biologic agents, and initial disease activity.

### Ethical statement

This study was conducted in accordance with the Declaration of Helsinki and Good Clinical Practice guidelines. The study protocol was approved by the institutional review board of the Jikei University School of Medicine (approval number: 33-422-11047). This study employed an opt-out approach for obtaining informed consent from all subjects and/or their legal guardians and providing the opportunity to decline participation.

## Results

### Thirty percent of patients without endoscopic healing was in symptomatic remission at the time of colonoscopy

Fifty-two patients received 53 biologic treatments over 12 weeks and underwent follow-up colonoscopies. Only 32 patients underwent colonoscopies within 1 year (60.4%), but all patients did within 2 years after starting biologic agents. Median time from drug initiation to follow-up colonoscopy was 58 weeks (IQR, 42.0–103.0 weeks). Thirty-three patients achieved EH within 2 years (62.3%). Table 1 shows the baseline characteristics of patients. Overall, 37 patients were treated with TNF-alpha agents (69.8%), 7 with VDZ (13.2%), and 8 with UST (15.1%). There was no statistically significant difference in the duration of biologic treatment between patients with EH and those without. The proportion of patients who received concomitant treatment with 5-ASA or thiopurines was not significantly different between both groups. A significantly greater number of patients with EH were in PRO2-based clinical remission and symptomatic remission than those without (PRO2-based clinical remission, 78.8 vs. 0.0%; symptomatic remission, 100.0 vs. 70.0%). None of patients without EH was in PRO2-based clinical remission, but 30% of them were in symptomatic remission despite a lack of EH. Among 33 patients with EH, 26 achieved complete EH (Mayo endoscopic sub-score [MES] 0, 78.8%).

**Table 1:** Baseline characteristics of patients.

	Endoscopic healing −	Endoscopic healing +	*P* value
Number of patients	20	33	
Age, mean (SD)	39.3 (18.8)	47.4 (15.5)	0.094
Gender, Male, n (%)	11 (55.0)	21 (63.6)	0.533
Disease location, Extensive colitis, n (%)	14 (70.0)	28 (84.9)	0.296
Extra-intestinal manifestation, n (%)	3 (15.0)	8 (24.2)	0.503
Biologics, n (%)			
IFX	0 (0.0)	5 (15.2)	
ADA	2 (10.0)	6 (18.2)	
GLM	11 (55.0)	13 (39.4)	
VDZ	2 (10.0)	5 (15.2)	
UST	5 (25.0)	3 (9.1)	
PRO2-based clinical remission, n (%)	0 (0.0)	26 (78.8)	< 0.001
Symptomatic remission, n (%)	14 (70.0)	33 (100)	0.002
Treatment duration, week, median [IQR]	51.5 [34.0–81.0]	62.0 [45.5–71.5]	0.420
Concomitant 5-ASA, n (%)	15 (75.0)	27 (81.8)	0.729
Concomitant thiopurines, n (%)	5 (25.0)	8 (24.2)	> 0.999
Complete endoscopic healing, n (%)	NA	26 (78.8)	

### Patients with endoscopic healing had a reduced risk of biologic treatment failure over time

**Figure 1:**
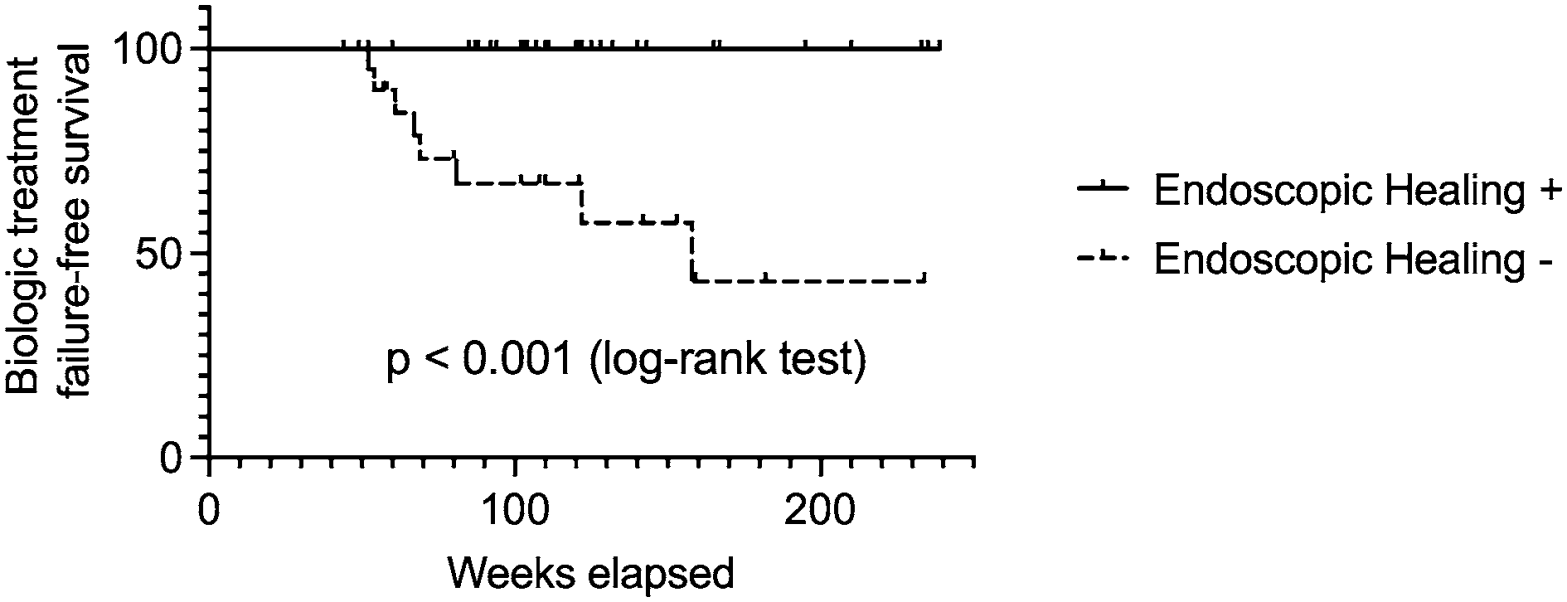
Kaplan–Meier survival analysis to evaluate the impact of endoscopic healing (EH) within 2 years of biologic initiation on treatment failure in patients with UC (N = 33 for EH+ and 20 for EH− subgroups).

During median follow-up period of 110 weeks after colonoscopies, all patients who achieved EH remained on biologic treatment without LOR. In contrast, 8 out of 20 patients who did not achieve EH experienced LOR and thereby discontinued treatment with biologic agents (relative risk = 3.75, 95% CI 2.23–5.51, *p* < 0.001). Median time from colonoscopy to drug discontinuation was 33 weeks (IQR, 12.0–69.0 weeks). There was no statistically significant difference in the time to colonoscopy and follow-up period after colonoscopy between both groups (Table 2). Kaplan–Meier estimator revealed a significantly lower risk of biologic treatment failure among patients who achieved EH within 2 years compared to those who did not (*p* < 0.001, log-rank test; Fig. 1). Similarly, patients who achieved EH within 1 year had a significantly lower risk of biologic treatment failure than those who did not (*p* < 0.05, log-rank test; Supplementary Fig. S1).

**Table 2:** Loss-of-response-related biologic treatment failure in patients with or without endoscopic healing.

	Endoscopic healing −	Endoscopic healing +	*P* value
Number of patients	20	33	
Biologic treatment failure, n (%)	8 (40.0)	0 (0.0)	< 0.001
Time to colonoscopy, week, median [IQR]	51.5 [34.0–81.0]	62.0 [45.5–71.5]	0.420
Follow-up period, week, median [IQR]	105.0 [62.5–150.3]	111.0 [90.0–141.5]	0.399

### Achievement of endoscopic healing was an independent factor associated with a reduced risk of biologic treatment failure

**Figure 2:**
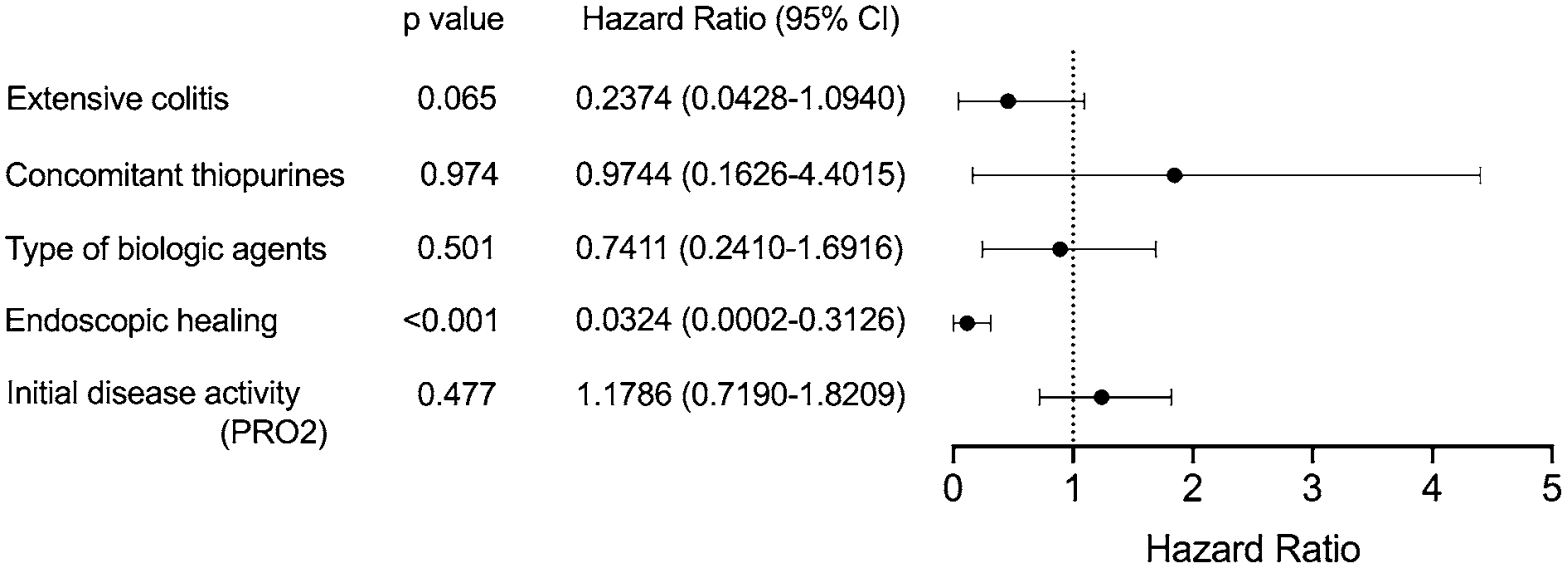
Cox regression analysis for loss-of-response-related biologic treatment failure.

To clarify the clinical characteristics of patients who experienced LOR-related biologic treatment failure, we compared the baseline characteristics at the time of colonoscopy between patients with biologic treatment failure and those without (Table 3). A significantly smaller number of patients with biologic treatment failure had extensive colitis than those without (50.0 vs. 84.4%, *p* = 0.048). A numerically smaller number of patients with biologic treatment failure received ADA treatment than those without (0.0 vs. 17.8%). There was no statistically significant difference in the proportion of patients who received concomitant treatments with 5-ASA or thiopurines. There was no statistically significant difference in the initial disease activity at the time of starting biologic treatment. The proportions of patients who were in PRO2-based clinical remission and symptomatic remission at the time of colonoscopy were significantly lower in patients with biologic treatment failure than in those without. However, half of patients with biologic treatment failure were in symptomatic remission at the time of colonoscopy. Among patients with biologic treatment failure, median time from colonoscopy to drug discontinuation was numerically shorter in those without symptomatic remission than those with (64.0 vs. 102 weeks, *p* = 0.34, Supplementary Fig. S2). A Cox regression analysis identified EH as an independent factor which is associated with lower risk of biologic treatment failure irrespective of disease extent, concomitant drugs, types of biologic agents, and initial disease activity (Hazard Ratio = 0.0324, 95% CI 0.0002–0.3126, *p* < 0.001; Fig. 2). Sensitivity analyses demonstrated the same results (Supplementary Fig. S3).

**Table 3:** Differences in the baseline characteristics between patients with biologic treatment failure and those without.

	Treatment failure	Non-failure	*P* value
Number of patients	8	45	
Age, mean (SD)	39.6 (13.7)	45.2 (17.6)	0.401
Gender, Male, n (%)	6 (75.0)	26 (57.8)	0.359
Disease location, Extensive colitis, n (%)	4 (50.0)	38 (84.4)	0.048
Extra-intestinal manifestation, n (%)	1 (12.5)	10 (22.2)	> 0.999
Biologics, n (%)			
IFX	1 (12.5)	5 (11.1)	
ADA	0 (0.0)	8 (17.8)	
GLM	5 (62.5)	19 (42.2)	
VDZ	1 (12.5)	6 (13.3)	
UST	1 (12.5)	7 (15.6)	
Concomitant 5-ASA, n (%)	7 (87.5)	35 (77.8)	> 0.999
Concomitant thiopurines, n (%)	2 (25.0)	11 (24.4)	> 0.999
Initial disease activity (PRO2), mean (SD)	1.88 (0.83)	1.27 (1.53)	0.069
PRO2-based clinical remission, n (%)	0 (0.0)	26 (78.8)	< 0.001
Symptomatic remission, n (%)	4 (50.0)	43 (95.6)	0.003
Endoscopic healing, n (%)	0 (100.0)	33 (73.3)	< 0.001

## Discussion

Achievement of EH is widely accepted as a long-term target of UC treatment, since it is associated with prolonged clinical remission and lower risks of disease-related complications^14,15^. Lots of clinical trials were designed to evaluate EH as an endpoint of biologic treatment for UC; however, whether achievement of EH is associated with the subsequent risk of LOR and treatment failure remains understudied. Using a Cox regression analysis, we demonstrated that achievement of EH is an independent factor associated with a reduced risk of LOR-related biologic treatment failure in patients with UC. Importantly, our study included patients with a variety of biologic treatment including IFX, ADA, GLM, VDZ, and UST, but this preferable impact of EH on the risk of biologic treatment failure was independent from the types of biologic agents or concomitant thiopurines.

Endoscopic assessment of mucosal disease activity after biologic initiation will provide a chance to adjust treatment for persistent mucosal inflammation and ultimately help patients achieve EH. However, the timing to perform a follow-up colonoscopy is highly dependent on the disease and treatment context of each patient and on each physician’s decision making because of lacking evidence-based predictors for clinical outcome. Using a commercial healthcare administrative claims database which include 7247 patients with UC initiating IFX, ADA, VDZ, or UST, Limketkai BN et al. reported that endoscopic evaluation within 1 year of drug initiation was associated with a lower overall risk of disease-related complications which include corticosteroid use, change of biologic agents, hospitalization, and surgery compared to the follow-up endoscopy after 1 year^16^. Although the character of database approach did not allow examining the impact of endoscopic findings on the risk of disease-related complications, their results implied the importance of adjusting biologic treatment based on endoscopic disease activity within 1 year of biologic initiation to obtain better clinical outcomes. In our study, achievement of EH within 2 years of biologic initiation was clearly associated with a reduced risk of LOR-related treatment failure. Half of patients who ultimately experienced LOR were in symptomatic remission at the time of follow-up colonoscopy but had to stop biologic treatment within the next 2 years. Remaining half of patients without symptomatic remission were at higher risk of LOR-related biologic treatment failure with shorter time to drug discontinuation. Although examining the best timing of follow-up endoscopy after biologic initiation is beyond the scope of this study, these results suggest that proactive evaluation of endoscopic activity within at least 2 years after biologic initiation might be helpful to find out patients with higher risk of LOR irrespective of the presence of symptomatic remission and provide them an invaluable opportunity to have treatment adjustment and stay on prolonged clinical remission.

There are several limitations in this study. First, our study is a retrospective cohort with a small number of patients and thus is exposed to a lot of biases. A multivariate analysis identified EH as an independent factor associated with a reduced risk of LOR-related biologic treatment failure. However, our results should be confirmed in a prospective cohort study with a larger number of patients in the future. Second, our study did not examine the difference in the risk of LOR-related treatment failure between EH within 1 year of biologic initiation and that within 2 years. This is beyond the scope of our study as we described above; however, the reduced risk of biologic treatment failure in patients with EH within 1 year implies that a follow-up colonoscopy within 1 year might be more preferable. Only 60% of patients underwent a follow-up colonoscopy within 1 year after biologic initiation in our study probably because most of the patients were symptomatically stable. Since abdominal symptom is not predictive enough for EH, more proactive endoscopy might be needed not to miss an opportunity for treatment adjustment. Third, we could not examine the difference in LOR-related biologic treatment failure between patients with complete EH and those with non-complete EH (MES 1). None of patients with complete or non-complete EH experienced LOR during the observational period, but a longer-term follow-up might be necessary to detect the difference since complete EH is known to be associated with superior disease outcomes compared to non-complete EH^9^. Similarly, we could not examine the effect of PRO2-based clinical remission on the risk of biologic treatment failure in patients who achieved EH. More than 20% of patients with EH were not in PRO2-based clinical remission at the time of follow-up colonoscopy but did not develop LOR to biologic agents during the observational period. Although EH is not always accompanied by complete normalization of stool frequency^17^, future studies with longer follow-up period might clarify the unknown impact of PRO2-based clinical remission on treatment outcomes.

In conclusion, in this retrospective cohort study, we identified EH as an independent factor associated with a reduced risk of LOR-related biologic treatment failure in patients with UC. A follow-up colonoscopy within 1–2 years of biologic initiation is recommended to adjust treatment for potentially persistent mucosal inflammation and stay on prolonged clinical remission.

### Supplementary Information

Supplementary Information.

## Data Availability

The datasets used or analyzed in this study are available from the corresponding author on reasonable request.

## References

[1] Kobayashi T (2020). Ulcerative colitis. Nat. Rev. Dis. Prim..

[2] Rutgeerts P (2005). Infliximab for induction and maintenance therapy for ulcerative colitis. N. Engl. J. Med..

[3] Sandborn WJ (2012). Adalimumab induces and maintains clinical remission in patients with moderate-to-severe ulcerative colitis. Gastroenterology.

[4] Sandborn WJ (2014). Subcutaneous golimumab maintains clinical response in patients with moderate-to-severe ulcerative colitis. Gastroenterology.

[5] Feagan BG (2013). Vedolizumab as induction and maintenance therapy for ulcerative colitis. N. Engl. J. Med..

[6] Sands BE (2019). Ustekinumab as induction and maintenance therapy for ulcerative colitis. N. Engl. J. Med..

[7] Savelkoul EHJ (2022). Systematic review and meta-analysis: Loss of response and need for dose escalation of infliximab and adalimumab in ulcerative colitis. Inflamm. Bowel Dis..

[8] Peyrin-Biroulet L (2019). Loss of response to vedolizumab and ability of dose intensification to restore response in patients with Crohn's disease or ulcerative colitis: A systematic review and meta-analysis. Clin. Gastroenterol. Hepatol..

[9] Turner D (2021). STRIDE-II: An Update on the selecting therapeutic targets in inflammatory bowel disease (STRIDE) initiative of the international organization for the study of IBD (IOIBD): Determining therapeutic goals for treat-to-target strategies in IBD. Gastroenterology.

[10] Jairath V (2015). Development of interim patient-reported outcome measures for the assessment of ulcerative colitis disease activity in clinical trials. Aliment. Pharmacol. Ther..

[11] Sandborn WJ (2020). Efficacy and safety of mirikizumab in a randomized phase 2 study of patients with ulcerative colitis. Gastroenterology.

[12] R Core Team. *R: A Language and Environment for Statistical Computing*. R Foundation for Statistical Computing (2020).

[13] Therneau, T. *A Package for Survival Analysis in R_. R package version 3.1–12* (2020).

[14] Reinink AR, Lee TC, Higgins PD (2016). Endoscopic mucosal healing predicts favorable clinical outcomes in inflammatory bowel disease: A meta-analysis. Inflamm. Bowel Dis..

[15] Shah SC, Colombel JF, Sands BE, Narula N (2016). Mucosal healing is associated with improved long-term outcomes of patients with ulcerative colitis: A systematic review and meta-analysis. Clin. Gastroenterol. Hepatol..

[16] Limketkai BN, Singh S, Jairath V, Sandborn WJ, Dulai PS (2019). US practice patterns and impact of monitoring for mucosal inflammation after biologic initiation in inflammatory bowel disease. Inflamm. Bowel Dis..

[17] Jharap B (2015). Randomised clinical study: Discrepancies between patient-reported outcomes and endoscopic appearance in moderate to severe ulcerative colitis. Aliment. Pharmacol. Ther..

