# Supplementary material for: Endoscopic healing is associated with a reduced risk of biologic treatment failure in patients with ulcerative colitis
Source: Sci Rep. 2024 Jan 3;14:303. doi: 10.1038/s41598-024-51208-2 (PMC10761669; doi:10.1038/s41598-024-51208-2)
Supplement: Supplementary file 1 — Supplementary Information. [file 41598_2024_51208_MOESM1_ESM.pdf]

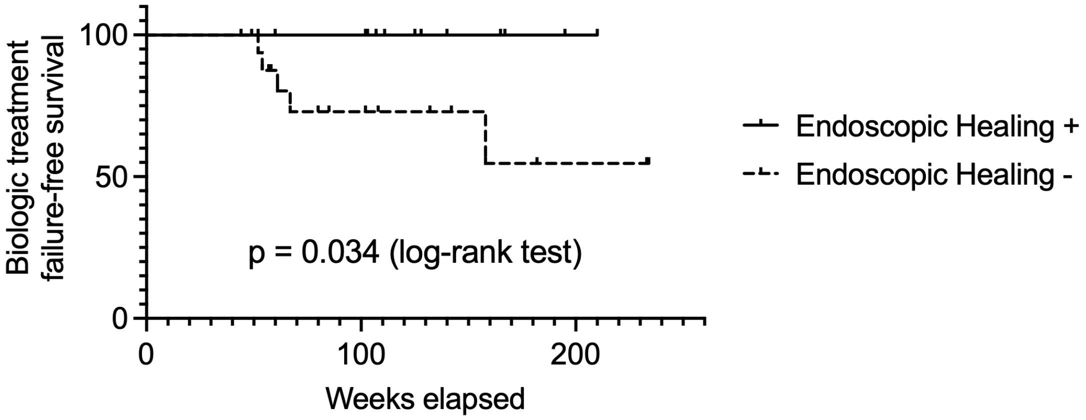

Supplementary figure 1. Kaplan–Meier survival analysis to evaluate the impact of endoscopic healing (EH) within 1 year of biologic initiation on treatment failure in patients with UC (N = 16 for each subgroup).

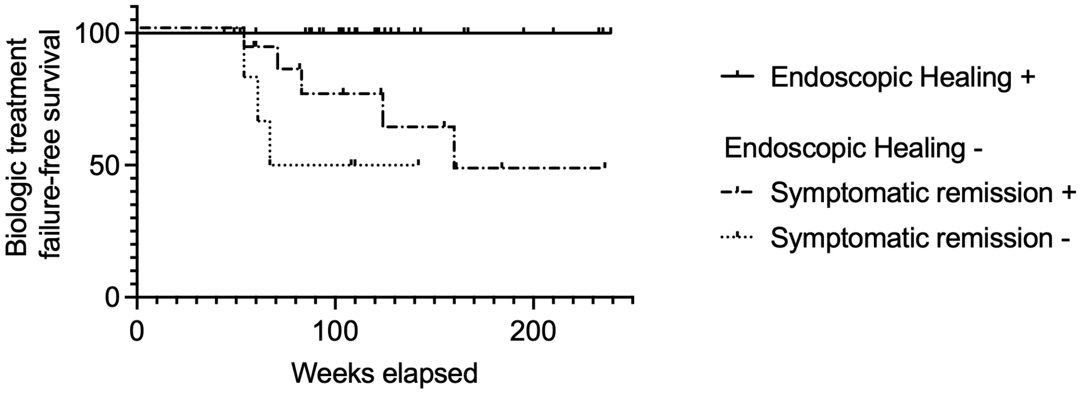

Supplementary figure 2. Kaplan–Meier survival analysis to evaluate the impact of endoscopic healing (EH) within 2 years of biologic initiation and symptomatic remission on treatment failure in patients with UC (N = 33 for EH+, 14 for EH- with symptomatic remission, and 6 for EH- without symptomatic remission subgroups).

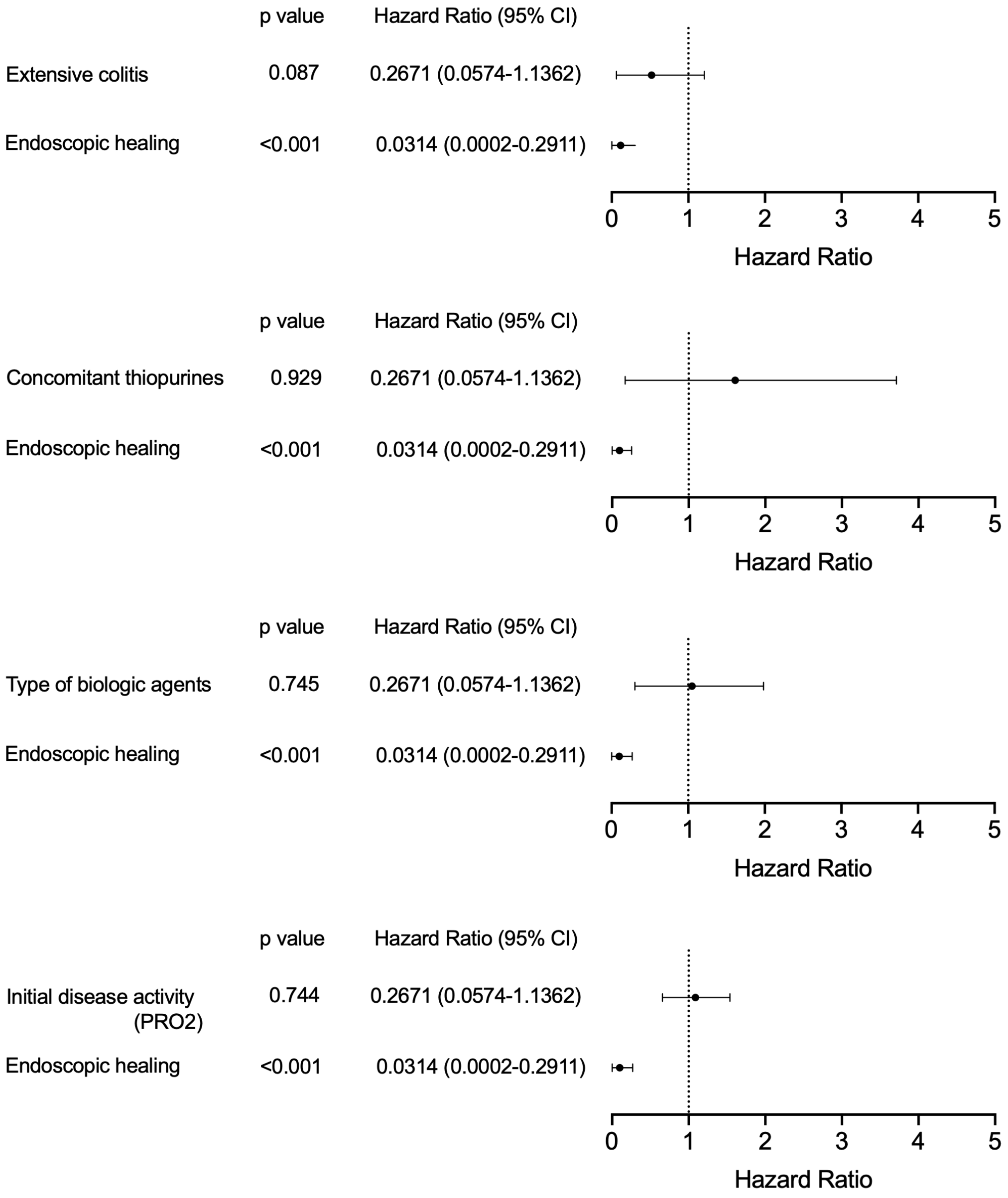

Supplementary figure 3. Cox regression analysis for loss-of-response-related biologic treatment failure.
